# Attitude of A Sample of Iranian Researchers toward The Future of Stem Cell Research

**DOI:** 10.22074/cellj.2018.5388

**Published:** 2018-05-28

**Authors:** Mahdi Lotfipanah, Fereydoon Azadeh, Mehdi Totonchi, Reza Omani-Samani

**Affiliations:** 1 Department of Medical Library and Information Science, Virtual School, Center for Excellence in E-Learning in Medical Education, Tehran University of Medical Sciences, Tehran, Iran; 2 Department of Epidemiology and Reproductive Health, Reproductive Epidemiology Research Center, Royan Institute for Reproductive Biomedicine, ACECR, Tehran, Iran; 3 Faculty of Allied Medical Sciences, Tehran University of Medical Sciences, Tehran, Iran; 4 Department of Genetics, Reproductive Biomedicine Research Center, Royan Institute for Reproductive Biomedicine, ACECR, Tehran, Iran

**Keywords:** Attitudes, Regenerative Medicine, Stem Cell, Treatment

## Abstract

**Objective:**

Stem cells that have unlimited proliferation potential as well as differentiation potency are considered to be a promising future treatment method for incurable diseases. The aim of the present study is to evaluate the future trend of stem cell researches from researchers’ viewpoints.

**Materials and Methods:**

This was a cross-sectional descriptive study on researchers involved in stem cell research at Royan Institute. We designed a questionnaire using a qualitative study based on expert opinion and a literature review. Content validity was performed using three rounds of the Delphi method with experts. Face validity was undertaken by a Persian literature expert and a graphics designer. The questionnaire was distributed among 150 researchers involved in stem cell studies in Royan Institute biology laboratories.

**Results:**

We collected 138 completed questionnaires. The mean age of participants was 31.13 ± 5.8 years; most (60.9%) were females. Participants (76.1%) considered the budget to be the most important issue in stem cell research, 79.7% needed financial support from the government, and 77.5% felt that charities could contribute substantially to stem cell research. A total of 90.6% of participants stated that stem cells should lead to commercial usage which could support future researches (86.2%). The aim of stem cell research was stipulated as increasing health status of the society according to 92.8% of the participants. At present, among cell types, importance was attached to cord blood and adult stem cells. Researchers emphasized the importance of mesenchymal stem cells (MSCs) rather than hematopoietic stem cells (HSCs, 57.73%). The prime priorities were given to cancer so that stem cell research could be directed to sphere stem cell research whereas the least preference was given to skin research.

**Conclusion:**

Regenerative medicine is considered the future of stem cell research with emphasis on application of these cells, especially in cancer treatment.

## Introduction

Stem cells are undifferentiated pluripotent cells that have the ability to renew themselves for an extended period of time or perhaps forever without changes in their properties. They can differentiate into other cell types (1). Stem cells are an important research field because of potential clinical applications and biological importance (2). According to recent stem cell researches, many believe that these cells could potentially cure a number of poor prognosis diseases like cancers or cardiovascular disease through cell-based replacement therapy (3). There are different sources of stem cells-embryonic (ES), adult, fetal, cord blood, and induced pluripotent stem (IPS) cells (4). Stem cell researches have focused on numerous areas such as gene therapy, tissue engineering, three dimensional (3D) cultures, production of recombinant proteins, and toxicology research (5). 

Alijani and Karami (6) searched for Iranian scientific publications that pertain to stem cells until 2007 that have been indexed in the Web of Science citation index. They classified these papers according to subjects, authors, research centers, impact factor, and international collaborations. Although a good study, this research could not ascertain researchers’ attitudes for the future. Li et al. (7) undertook a similar study in which they reviewed stem cell papers from 1996 to 2006 in the Web of Science citation index. They included the type of stem cells, conditions and diseases studied. In 2015, a paper was published about the landscape of cell therapy in the UK (8) which pointed to the areas of cancer, cardiology, and neurology as the most frequent for trials although most of the included trials were in the early phases. Luo and Matthews (9) studied stem cell papers from 2000 to 2010 and the changes during this period of time. They focused on the countries and international collaborations but did not focus on cell types, conditions, or diseases. None of the previous studies have focused on the future trends of stem cell research. The trend of stem cell researches varies according to different contexts. This trend has a direct relationship to the attitude of the researchers, research center directors, and policy makers. We have sought provide answers about the future trend of stem cell researches. Therefore, we designed this study to evaluate the attitudes of stem cell researchers toward the future trend of stem cell research in Iran.

## Materials and Methods

In this was a cross-sectional descriptive study, we chose Royan Institute for Stem Cell Research for this study. Royan Institute established the first human ES cell line (10) and is considered one of the most active research centers in this field. 

We gathered the opinions of researchers who have worked in the stem cell laboratories for at least one year and had either a temporary or permanent contract with Royan Institute. We considered all academic staff, researchers, and Ph.D. students to be potential participants. We distributed 150 questionnaires to be anonymously completed by these participants. 

We performed a qualitative study that pertained to the trend of stem cell research and conducted in-depth interviews with 12 experts in cell biology and research directors. We extracted the items from the interviews. In addition, a number of items were added after a literature review. The items were changed to questions followed by a three-round Delphi to assess for content validity of the questionnaire. The first round had 40 contributors, which was subsequently reduced to 18 contributors in the final round. The final questionnaire was assessed for face validity by a Persian literature expert and designed by a graphic expert. The final questionnaire contained 23 questions in the following 9 categories: budget, aims, cell type, organ or disease, research field, regenerative medicine, international collaborations, cell therapy, and time of application of cells. A total of 8 questions were completed with a Likert scale (completely disagree to completely agree), 3 questions pertained to the types of cells, 3 questions about priorities that should be ordinary, one question about the collaborating countries, and the rest pertained to questions about timing.

### Statistical analysis 

Statistical analyses were carried out using the SPSS statistical software package (SPSS Inc., Chicago, IL, USA) version 22.0. Continues variables were expressed as mean ± SD and categorical variables as numbers (percentages). Normality of the variables was checked by the Kolmogorov-Smirnov test. We used the independent sample’s t test and one-way analysis of variance (ANOVA) followed by Tukey’s post hoc test for numerical variables and the chi-square test for categorical variables. P<0.05 was considered statistically significant.

### Ethical considerations

We assured participants that they were free to participate and none of their personal data would be published. All questionnaires were completed anonymously and voluntarily completing the questionnaire was considered consent. 

## Results

Of the 150 distributed questionnaires, we retrieved 138 completed ones with a response rate of 92%. Participants had a mean ± SD age of 31.13 ± 5.8 years; 60.9% were female. Only 10.9% of our participants were academic staff and the remainder consisted of researchers.

### Budget

A total of 76.09% of participants considered the budget as the most important issue in stem cell research compared to 14.49% who disagreed and 9.42% who had no opinion. The essential role of government financial support was supported by 79.7% compared to 11.6% who disagreed and8.7% who had no idea. There were 77.5% of participantswho agreed with financial support of the researches bycharities, 9.4% disagreed and 13% had no idea. Stem cellproducts, as a financial source, were supported by 86.2%, 2.9% disagreed and 10.9% had no idea. Overall agreementwith commercialization of stem cell products that resultedfrom researches was 90.6%, whereas 2.9% disagreed and6.5% had no idea. We performed quantitative analysis withcomplete disagreement considered as "1" and completeagreement as "5". The results showed the following mean ± SD of participants’ ideas about the budget as the main issue (3.85 ± 0.99), governmental support (3.91 ± 0.88), support bycharities (3.88 ± 0.83), stem cell products as research support (4.21 ± 0.75), and overall commercialization of stem cell products (4.34 ± 0.76).

### Main aim of researches

**Fig.1:**
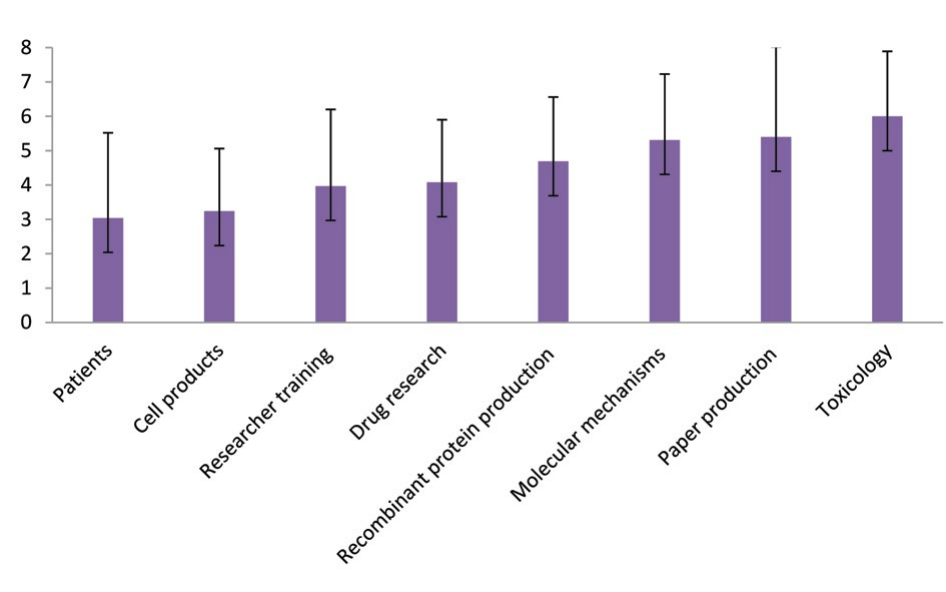
Stem cell research priorities.

A total of 81.2% agreed with increasing the prestige andscientific credibility of the country, whereas 6.5% disagreedand 11.6% had no idea. Increasing health level of the societywas supported by 92.8%, 2.2% disagreed and 5.1% had noidea. Stem cell research priorities (lowest 8^th^ and highest 1^st^) were as follows (mean ± SD): application on patients (3.04± 2.48), cell products (3.24 ± 1.82), researcher training (3.97± 2.23), drug researches (4.08 ± 1.82), recombinant proteinproduction (4.69 ± 1.87), molecular mechanisms (5.31 ± 1.92), paper production (5.40 ± 2.62), and toxicology (6.00 ± 1.89, Fig .1). 

### Cell types-priority of stem cell types and sources 

**Fig.2:**
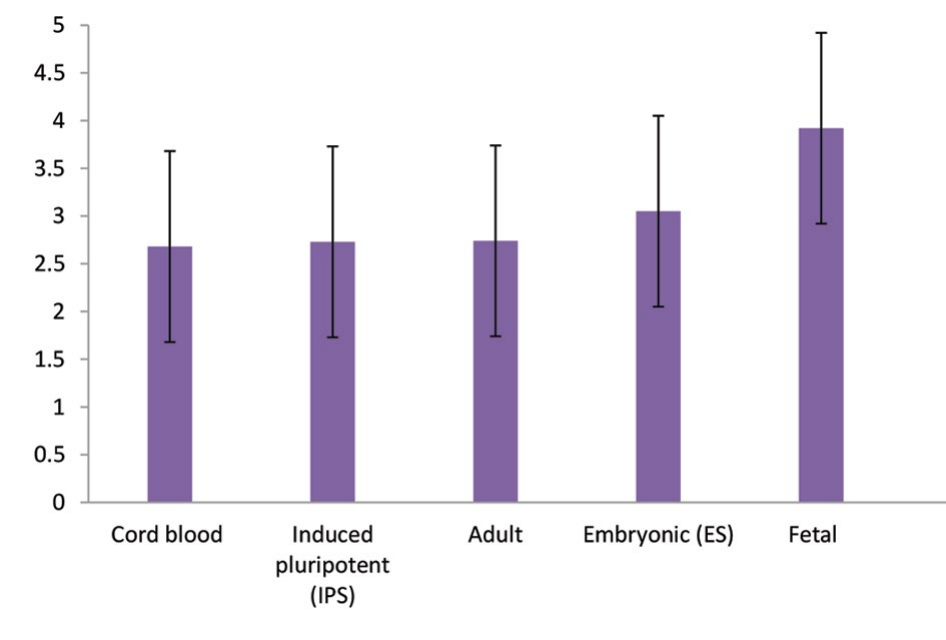
Priority of stem cell types and sources.

**Fig.3:**
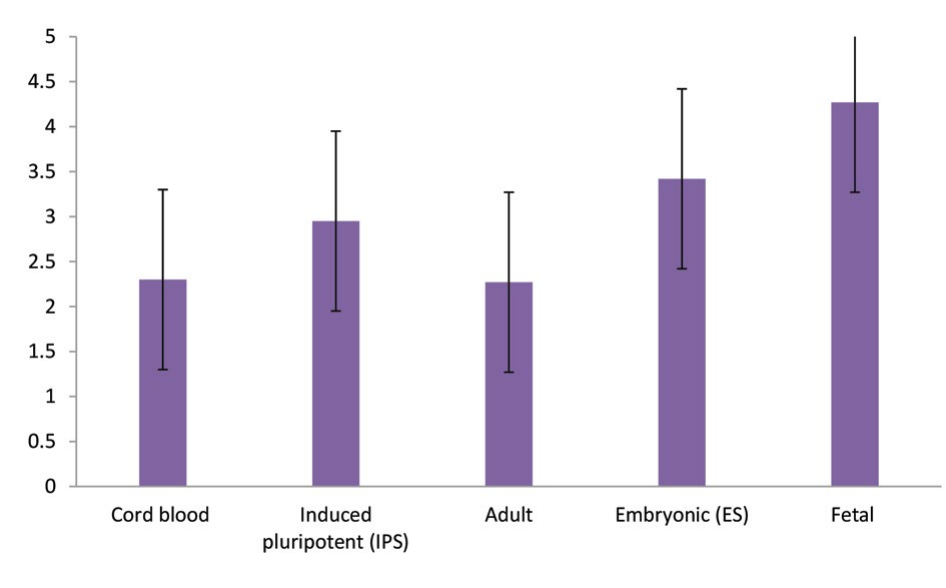
The remaining time (years) for each cell type for use in routine treatment.

Participants prioritized the answers as follows: (lowest priority 5^th^ and highest priority 1^st^) cord blood stem cells (2.68 ± 1.14), IPS (2.73 ± 1.42), adult stem cells (2.74 ± 1.56), ES (3.05 ± 1.39), and fetal stem cells (3.92 ± 1.19). The ANOVA test determined that a significant difference existed between these priorities (F=19.24, P<0.001, Fig .2). The time remaining for each cell type for usage in routine treatment (minimum 1^st^ and maximum 5^th^) was reported as follows: adult stem cells (2.27 ± 1.51), cord blood stem cells (2.3 ± 1.15), IPS (2.95 ± 1.36), ES (3.42 ± 1.21), and fetal stem cells (4.27 ± 4.31, Fig .3). ANOVA showed a significant difference between these priorities (F=17.45, P<0.001). Among the two types of adult stem cells, mesenchymal stem cells (MSCs) and hematopoietic stem cells (HSCs), the percentage of focus on MSCs was 51.67% compared to HSCs (48.33%) which significantly differed (T=5.615, P<0.001). 

### Organ or disease priorities: priority of body organs, systems or diseases 

**Fig.4:**
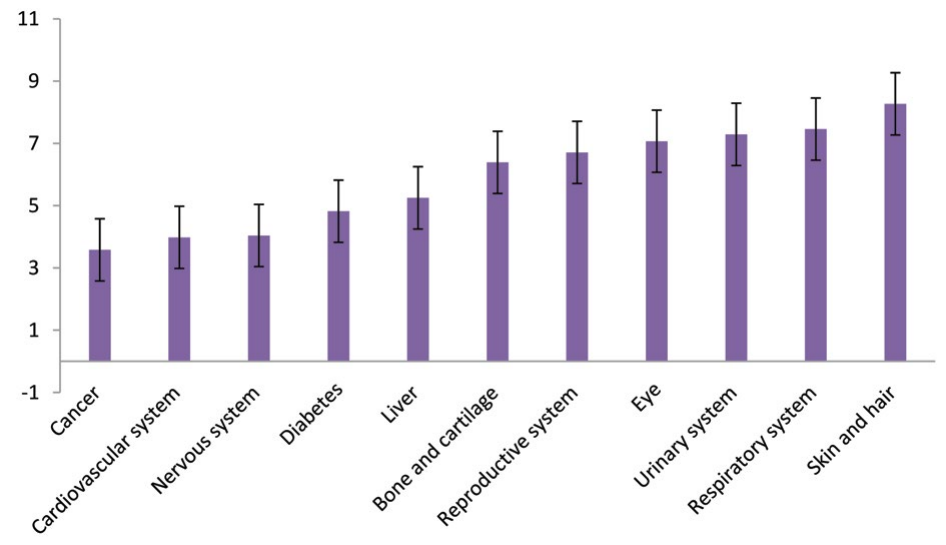
Organ or disease priorities.

These were (minimum 11^th^ and maximum 1^st^) as follows: cancer (3.58 ± 3.17), cardiovascular system (3.98 ± 2.47), nervous system (4.04 ± 2.98), diabetes (4.82 ± 2.8), liver (5.25 ± 2.69), bone and cartilage (6.39 ± 2.95), reproductive system (6.71 ± 3.69), eyes (7.07 ± 2.65), urinary system (7.29 ± 2.68), respiratory system (7.46 ± 2.54), and skin and hair (8.27 ± 2.87, Fig .4).

### Research field priority 

The mean ± SD results of researchers’ ideas about priority of research fields (minimum 0 and maximum 6) were: gene therapy (3.9 ± 1.76), tissue engineering (3.82 ± 1.76), recombinant proteins (3.51 ± 1.49), trans- differentiation (3.40 ± 1.88), 3D culture (3.39 ± 1.56), and bio-molecular systems (3.36 ± 1.69). There was no significant difference between these ideas.

### Regenerative medicine as the future road map of the stem cell researches 

A total of 80.43% of participants agreed with this item, 3.62% disagreed, and 15.94% had no idea. 

### International collaborations: Reaction to international collaboration as the most important factor for development of stem cell science 

There were 79.71% of participants who agreed, 5.80% disagreed and 14.49% had no idea. Responses to the best geographic area for scientific collaboration were as follows (minimum 5^th^ and maximum 1^st^): USA (1.77 ± 1.15), Europe (1.91 ± 0.84), Australia (3.5 ± 0.87), Eastern Asia (3.51 ± 1.06), and the Middle East (4.32 ± 1.13).

### Cell therapy instead of drug therapy: Responses to substitution of cell therapy for current drug therapy in the future 

A total of 50% of participants agreed, 31.62% disagreed and 18.38% had no idea. 

### Time of application of each stem cell type in treatment of disease 

The maximum response to application times for ES cells was 10 years (43.5%), currently for adult stem cells (34.8%), currently for cord blood stem cells (44.9%), 5 years for fetal stem cells (29%), and 5 years for IPS (31.5%, Table 1). 

**Table 1:** Timing of stem cell applications in current treatments


Cell type	Current	Within 5years	Within 10years	Within 20years	Never	Total

First clinical trial on embryonic stem (ES) cells	25 (18.93%)	63 (47.72%)	35 (26.51%)	5 (3.78%)	4 (3.03%)	132
Application of ES cells in treatment	7 (14.28%)	38 (28.57%)	60 (45.11%)	20 (15.03%)	8 (6.01%)	133
Application of adult stem cells in treatment	48 (36.92%)	35 (26.92%)	33 (25.38%)	12 (9.23%)	2 (1.53%)	130
Application of cord blood stem cells in treatment	62 (46.26%)	39 (29.1%)	22 (16.41%)	10 (7.46%)	1 (0.74%)	134
Application of fetal stem cells in treatment	12 (9.37%)	40 (31.25%)	33 (25.78%)	28 (21.87%)	15 (11.71%)	128
Application of induced pluripotent stem (IPS) cells in treatment	15 (11.27%)	44 (33.08%)	41 (30.82%)	27 (20.3%)	6 (4.51%)	133


Data are presented as number (%).

## Discussion

We noted that participants mentioned budget as the most important issue in stem cell researches. Trounson and DeWitt (11) previously reported this finding. Budget has appeared to be the main issue worldwide. Investment in these researches in the United States is as follows: California (300 million dollars), Connecticut (10 million dollars), Maryland (14 million dollars), and New York (55 million dollars) (12). In March 2009, former President Obama has stated: "(m)edical miracles do not happen simply by accident. They result from painstaking and costly research and so on and from a government willing to support that work" (13). His statement clearly showed the importance of financial support in the stem cell field. However, support of the researches by charities has been a main issue throughout the world (14). This item has been considered to be one of the main solutions for a research budget. On the other hand, commercialization seems to be the most practical way to support stem cell researches. Weinryb and Bubela (14) in a study of California, Sweden, and South Korea have found that all three types of financial support (government, charities, and commercialization of stem cell products) were necessary for progress in this field. Other researchers confirmed this finding (15). Although commercialization has been mentioned as the most practical way to obtain a budget, it also has some consequences. It has been stated that benefits of commercialization are adequate to cover the complications and there are ways to manage the consequences (16). Good supervision with adequate laws, legislation and guidelines appears to lead to commercialization (17, 18).

The main aim of stem cell researches according to our participants was its application for treatment of diseases. As far as we could determine, no other study examined the opinion of the researchers about this matter.

Adult stem cells were considered to be the first priority followed by IPS. Monsarrat et al. (19) reviewed registered stem cell based trials and reported that 51% of these trials used MSCs. Our data showed that among adult stem cells, MSCs were a priority compared to HSCs. Bisson et al. (8) reported the same results for MSCs in UK trials as did other trials (20). The place of IPS cells became higher than the previously prominent ES cells (21). Seres and Hollands considered cord blood stem cells to be the future of regenerative medicine (22); however, the current study participants did not agree. In this research center there are both public and private cord blood banks (23). 

The current study data showed that cancer was the most important subject for stem cell researches followed by cardiovascular problems. Monsarrat et al. (19) reported that the highest number of stem cell trials pertained to the field of the cardiovascular system. Our participants chose the skin as the last priority, however they stated that the eyes were the last priority. The reason could be the numerous successful trials on the eyes in Royan Institute. The first successful clinical trial in this center was performed on corneal injuries (24). Li et al. (7) confirmed the results reported by Monsarrat et al. (19) that revealed the cardiovascular system, nervous system, and cancer as the three main fields for stem cell based trials. Our participants put the cardiovascular system after cancer as the second priority. Bisson et al. (8) showed concordant results of the cell based trials from all around the world. Gene therapy was considered the top research field in stem cell researches, whereas among cell based trials, differentiation was the top research field (25). This could be attributed to recent improvements in the field of gene therapy worldwide (26), which has changed the trend of future researches in this field. Currently, studies use gene therapy with stem cells such as HSCs (27), fetal stem cells (28), MSCs (29), and IPS (30). 

Our participants have stated that “regenerative medicine” is the future of medical treatments, which is a finding that appears to be a global idea (31). Half of the current study participants believe cell therapy will replace drug therapy in the future. 

The researchers have stated that ES cells will be used in routine treatments in 10 years. Trounson and DeWitt (11) have predicted that ES cells will be used in 3-6 years for eye treatments, 5 years for spinal cord injuries, and more for diabetes and cardiovascular diseases. Gearhart predicted that ES cells would be used as treatments in a few years (32). According to the current study participants, some of these cells are currently used in practice which are supported by other studies such as adult (33) and cord blood (34) stem cells. There are more than 40000 HSC transplantations annually in Europe (33).

International collaboration was considered an important issue in this field of research. Previously, higher citations of papers with multi-national authors have been reported (35, 36). The United States was considered to be the best place for international collaboration, which could be due to the large numbers of research papers in this field from the United States (37). 

## Conclusion

Future trends of stem cell research in Iran would focus more on cancer and the cardiovascular system. Application of these cells, particularly adult stem cells and IPS cells, will be the most important aim of stem cell researches. Iranian researchers are enthusiastic to work in multicenter multidisciplinary studies with international collaboration.
